# Supplementary figures and images for: Comparative Study of the Expression Profiles of miRNAs of Milk-Derived Exosomes of Yak and Jeryak
Source: Animals (Basel). 2022 Nov 17;12(22):3189. doi: 10.3390/ani12223189 (PMC9686552; doi:10.3390/ani12223189)

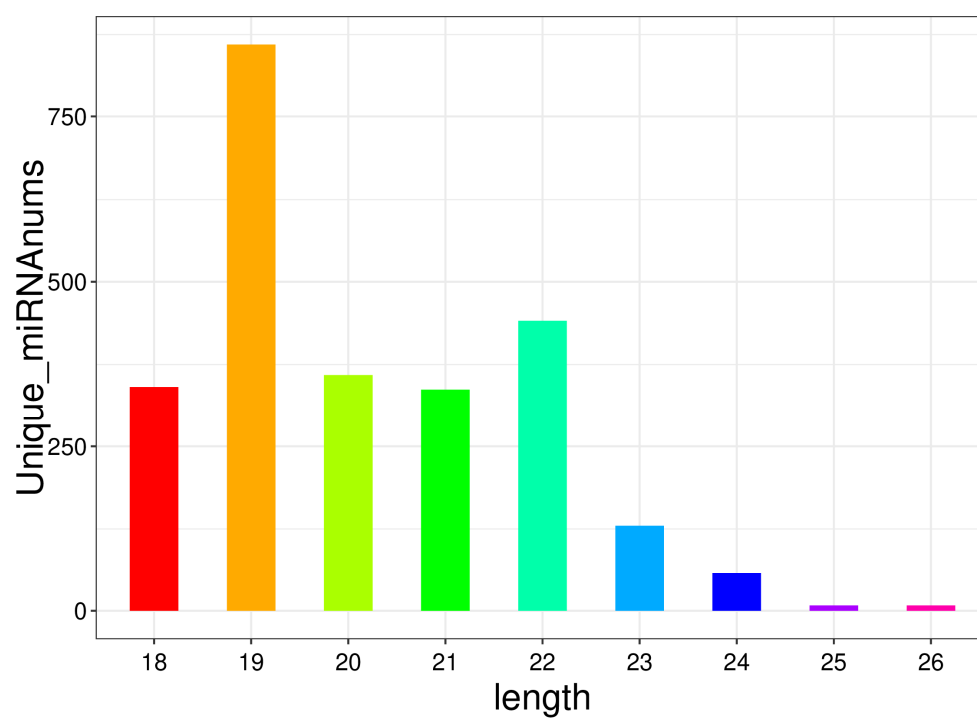

**Figure S1.** length distribution of miRNAs.

Supplement: Supplementary file 1 [file animals-12-03189-s001.zip › Supplementary materials/Figure S1. length distribution of miRNAs..pdf]

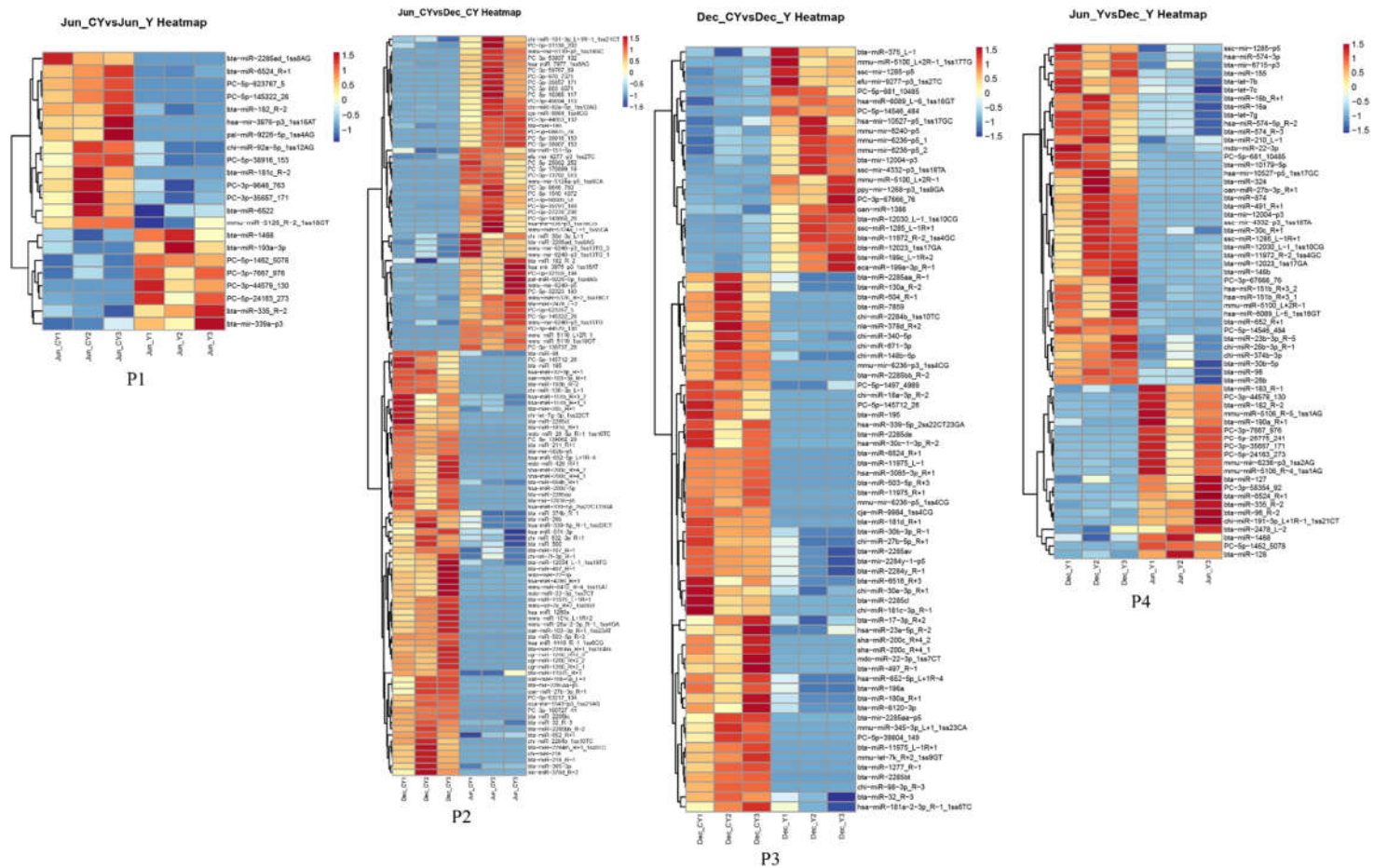

Figure S2. Heat map of DEMs.

Supplement: Supplementary file 1 [file animals-12-03189-s001.zip › Supplementary materials/Figure S2. Heat map of DEMs..pdf]
